# Metabolomic analysis of primary human skeletal muscle cells during myogenic progression

**DOI:** 10.1038/s41598-020-68796-4

**Published:** 2020-07-16

**Authors:** Ashok Kumar, Yashwant Kumar, Jayesh Kumar Sevak, Sonu Kumar, Niraj Kumar, Suchitra Devi Gopinath

**Affiliations:** 1 Translational Health Science and Technology Institute (THSTI), Faridabad, India; 2 Non-communicable Disease (NCD), Translational Health Science and Technology Institute (THSTI), NCR Biotech Science Cluster, 3rd Milestone, Faridabad-Gurugram Expressway, PO box #04, Faridabad, 121001 India; 3 Multi-Clinical Translational Research Center, Translational Health Science and Technology Institute (THSTI), NCR Biotech Science Cluster, 3rd Milestone, Faridabad-Gurugram Expressway, PO box #04, Faridabad, 121001 India; 4 Pediatric Biology Center, Translational Health Science and Technology Institute (THSTI), NCR Biotech Science Cluster, 3rd Milestone, Faridabad-Gurugram Expressway, PO box #04, Faridabad, 121001 India

**Keywords:** Biological techniques, Cell biology

## Abstract

Skeletal muscle constitutes more than 30% of total body mass using substrates such as glycogen, glucose, free fatty acids, and creatinine phosphate to generate energy. Consequently, multinucleated myofibers and resident mononucleated stem cells (satellite cells) generate several metabolites, which enter into circulation affecting the function of other organs, especially during exercise and atrophy. The present study was aimed at building a comprehensive profile of metabolites in primary human skeletal muscle cells during myogenic progression in an untargeted metabolomics approach using a high resolution Orbitrap Fusion Tribrid Mass Spectrometer. Identification of metabolites with multivariate statistical analyses showed a global shift in metabolomic profiles between myoblasts undergoing proliferation and differentiation along with distinctly separable profiles between early and late differentiating cultures. Pathway analyses of 71 unique metabolites revealed that Pantothenate metabolism and Coenzyme A biosynthesis and Arginine Proline metabolism play dominant roles in proliferating myoblasts, while metabolites involved in vitamin B6, Glyoxylate and Dicarboxylate, Nitrogen, Glutathione, and Tryptophan metabolism were upregulated during differentiation. We found that early and late differentiating cultures displayed differences in Phenylalanine, Tyrosine, Glycine, Serine and Threonine metabolism. Our results identify metabolites during maturation of muscle from progenitor myoblasts that have implications in muscle regeneration and pathophysiology.

## Introduction

Skeletal muscle physiology is critically dependent on the functionality of a progenitor population called, “satellite cells” that proliferate and differentiate to form multinucleated myofibers, thereby contributing to muscle regeneration during injury or exercise^1–4^. Conversely, defects in satellite cell function can result in loss of muscle mass and decline in performance that is frequently observed in chronic illnesses and aging^5^. In this aspect, the field of metabolomics has emerged with the intention of providing a comprehensive profile of metabolites and low molecular weight molecules in specific organ tissues, thereby enabling precision medicine and biomarker discovery in various disease states^6^. Thus, identification of metabolic pathways during myogenic progression may provide information on energy requirements of cells during various physiological states of the muscle tissue.

Recent studies have characterised the skeletal muscle metabolome during strenuous exercise in humans, neuromuscular diseases such as Pompe disease and Duchenne’s muscular dystrophy, daily variations in tissue metabolites vis-à-vis nutritional challenges, overexpression of metabolic regulators, and aging^7–12^. Metabolic profiles of young, post-mortem, and aging murine satellite cells have also been evaluated using measurement of mitochondrial function and analysis of metabolic gene signatures associated with different myogenic cell cycle states ^13,14^. Thus, studies from murine myogenic cells suggest that metabolic requirements are critically linked to myogenic cell fates and therefore the quality of regeneration. While the use of animal models has hugely aided in developing personalized metabolic profiles in each condition, few studies have used human subjects or cell culture models to achieve similar objectives. For such studies, muscle biopsies have been extracted from control and test subjects including serum samples to detect circulating metabolites and expand the repertoire of metabolites associated with a specific physiological state^9,10^ . Myogenic cell culture studies, on the other hand provide direct evidence of the presence of muscle-specific metabolites without confounding results arising from inter-tissue communication and endocrine regulators targeting skeletal muscle^7,15^. The objective of our study, therefore, was to develop a standard metabolomic profile of human primary myoblasts using ultra-high performance liquid chromatography tandem mass spectrometry (UHPLC-MS/MS) coupled to multivariate statistical analyses during different stages of myogenic progression, which can then be used for identifying signature metabolic profiles of skeletal muscles in altered physiological states.

## Results

### Metabolomic analysis

**Figure 1:**
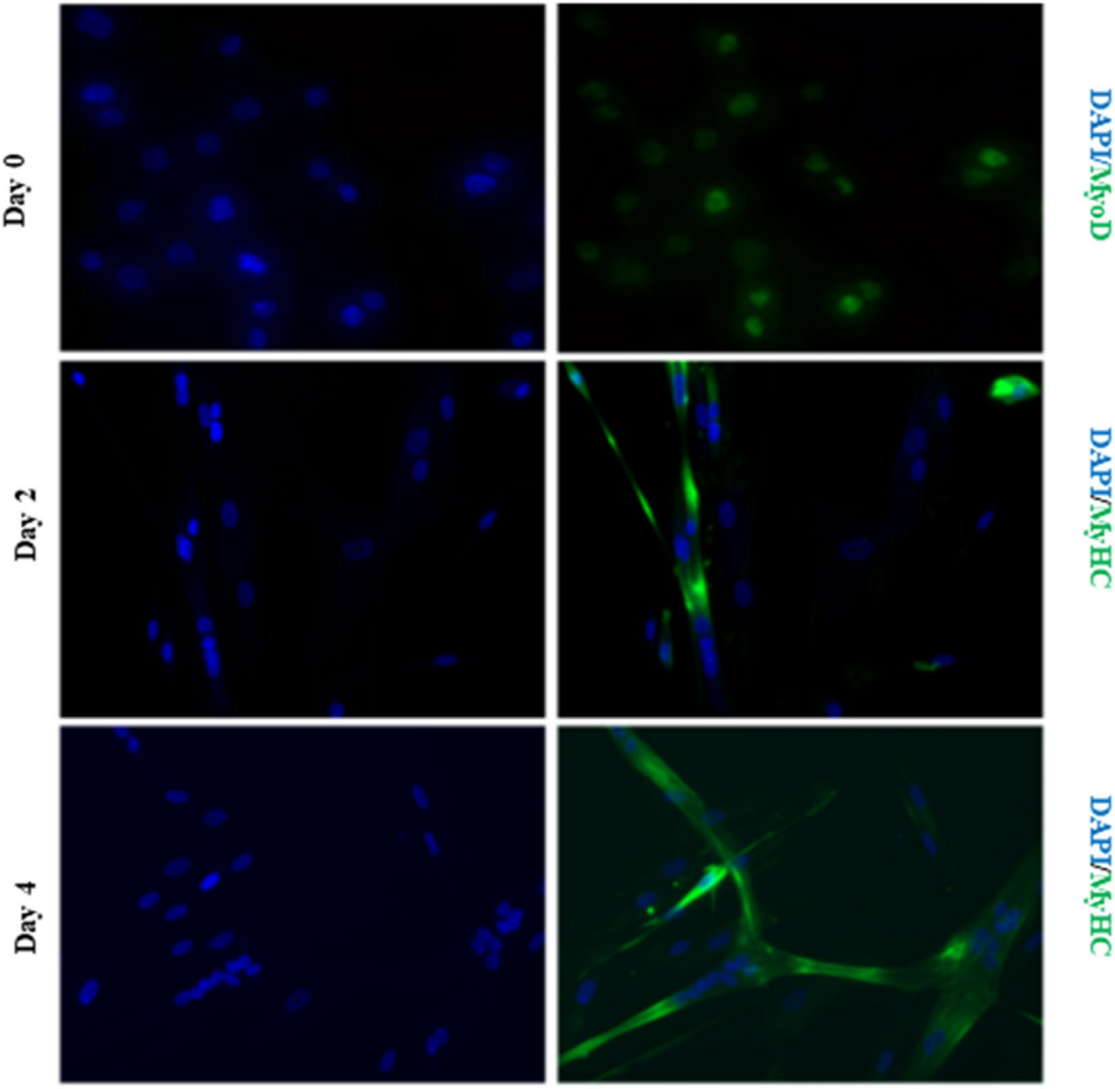
hSKmc cultured in growth media for 3 days were fixed and stained with MyoD (top panel), or switched to differentiation media for 2 days (middle panel) and 4 days (last panel) and stained for MyHC.

Cells lysates were derived from proliferating primary hSkmc at (Day 0) and differentiating HSMM cultures at two different days after induction of differentiation (Day 2 and Day 4) and were analyzed using UHPLC-MS/MS. Metabolites were identified and then analyzed using PCA analysis to obtain general information of the datasets, relationships between the different groups, and the detection of outliers (located outside the 95% confidence region of the model) (Supplementary Fig. 1B). Of the total 71 metabolites that were detected, principal component PC2 effectively and distinctly separated the Day 0 and Day 2/4 cultures suggesting that the switch from proliferation to differentiation causes a significant change in global metabolite profiles (Supplementary Fig. 1B). Proliferating myogenic cultures (Day 0) displayed greater variation as evidenced by the spread along the PC1 axis, possibly due to heterogeneity arising from asynchronous cell cycle times amongst cells. Early differentiating cultures (Day 2) while distinct from proliferating myoblasts, still showed considerable variation probably due to cells transiting from proliferation to differentiation. This is evident by the presence of mononucleated and multinucleated cells in the culture (Fig. 1, middle panel). Late differentiating cultures (Day 4), on the other hand, were represented by a tight cluster indicating culture homogeneity and increased presence of multinucleated cells (Fig. 1, Supplementary Fig. 1B). Gene expression analysis of known myogenic genes, MyoD, Myogenin, and Myosin Heavy Chain (MyHC) were activated as reported before during proliferation, early and late differentiation respectively (Supplementary Fig. 1A). Visualization of data using heat map analysis corroborated the distinction in PCA analyses between metabolite patterns of proliferating, early and late differentiating cultures (Supplementary Fig. 2).

Of the 71 identified unique metabolites, approximately 60% of the metabolites fell in the category of amino acids or derivatives and metabolites of amino acids. Of the rest, 18% of the metabolites were represented by carboxylic acids, 8% by nucleosides, 4% by esters, 3% by aldehyde and their derivatives, and less than 2% by carbohydrate polymers, alkaloids, and purine derivatives (Table 1).

**Table 1:** Global and differential metabolite profiles during myogenic progression of primary human myoblasts.

S. no	Metabolite name	Mode	Day-0/ versus Day-2		Day-0/ versus Day-4		Day-2/ versus Day-4	
			Ratio	*p* value	Ratio	*p* value	Ratio	*P* value
1	3-Methylhistidine	Positive Hilic	1.957483761	*0.01705873*2	1.969849069	*0.01706663*	1.482804653	0.956364752
2	5-Hydroxytryptophan	Positive Hilic	0.892634278	0.501064879	0.990409135	0.857467453	0.539920534	0.391557516
3	Arginine	Positive Hilic	2.063405284	*0.021845753*	3.019269217	0.129708384	64.10815532	0.510461226
4	Carnosine	Positive Hilic	1.381165782	0.231663886	0.441840836	0.388254784	0.925133238	0.326132666
5	Galactosamine	Positive Hilic	2.400296514	*0.011032868*	3.063427592	*0.014300249*	0.063095683	0.055447395
6	Glutathione (oxidized)	Positive Hilic	0.155939966	*0.008695074*	0.140495964	*0.040022369*	18.45594754	0.451109586
7	Glutathione (reduced)	Positive Hilic	0.219887217	0.478180308	0.508236024	0.371266079	47.07290561	0.653463664
8	Gly-Val	Positive Hilic	2.618389502	0.265807903	2.610573581	0.239896174	0.06066872	0.980335803
9	Histidine	Positive Hilic	0.897460993	0.824289642	0.68752904	*0.041119919*	5.333627958	0.590077911
10	Kynurenine	Positive Hilic	1.403423107	0.206203297	1.877591002	*0.045644309*	0.313956092	0.206462409
11	Lysine	Positive Hilic	1.07404302	0.69878706	1.262601926	0.439139483	41.26193431	0.370038949
12	*N*-Acetylhistidine	Positive Hilic	8.663894138	*0.010640991*	12.71642777	*0.011309978*	0.043353965	0.166758226
13	l-Methylhistidine	Positive Hilic	1.146680443	*0.006762015*	0.909198318	*0.088270644*	1.191416337	*0.015088396*
14	*N*-Methylisoleucine	Positive Hilic	0.883831782	0.656091485	1.693482061	0.733860783	2.87311408	0.690666886
15	*N*-Methylproline	Positive Hilic	1.434443191	0.356711878	2.093768751	*0.288915143*	1.250040637	0.208222283
16	*N*-Methylserine	Positive Hilic	0.630955667	0.050240636	0.51163564	0.019520417	21.3565562	0.250600044
17	Octopine	Positive Hilic	5.287217325	0.197774148	12.78205406	0.172678835	0.738545396	*0.019932737*
18	Proline–hydroxyproline	Positive Hilic	1.699381081	*0.015190354*	2.008944192	*0.033992985*	1.515058751	0.174178233
19	Propionylcarnitine	Positive Hilic	1.178580994	0.131295737	0.804876763	0.7130271	11.61778227	0.530650712
20	SDMA	Positive Hilic	2.317179658	0.429051246	3.662975851	0.304548183	0.005772778	0.300783066
21	Taurine	Positive Hilic	1.152807858	0.378443043	1.135080404	0.532004759	0.724821458	0.939454594
22	Uridine-5-diphosphoacetylglucosamine	Positive Hilic	0.236797735	0.285847398	0.320233968	0.203784286	332.6518156	0.70071203
23	Beta-alanine	Positive Hilic	2.343509429	*0.009954229*	2.496581273	*0.056740483*	0.100688392	0.820501657
24	Glycine	Positive Hilic	0.46210005	0.16294956	0.487130168	0.056597298	2.098289127	0.766787981
25	Palmitate	Positive Hilic	0.614986594	0.464993079	1.090262794	0.880114504	0.002434426	0.600636379
26	Cholate	Positive Hilic	1.098018515	0.157506917	1.094544916	0.364194308	13.14097994	0.977100429
27	Glycerophosphocholine	Positive Hilic	0.410966821	*0.028671262*	0.361175125	0.173575736	9.097481347	0.726250188
28	Glutamate	Positive Hilic	1.221537042	0.207605635	1.025410416	0.690595173	2.45540182	0.176857341
29	Leucine	Positive Hilic	1.444908173	0.176917283	1.728757289	0.275269829	10.29942336	0.483717136
30	4-Quinolinecarboxylic acid	Negative Hilic	1.418438368	0.513853945	2.159369267	0.218075199	0.103449101	0.086485699
31	Aspartic acid	Negative Hilic	0.482476444	0.065825708	0.602504147	0.113049977	0.127367313	0.516568913
32	Glutamic acid	Negative Hilic	1.046514892	0.780023849	0.827811878	0.242537341	0.150792733	0.415386587
33	Ornithine	Negative Hilic	1.850187949	0.391006337	5.755406751	0.145940321	0.078217893	0.165848257
34	Pyroglutamic acid	Negative Hilic	1.367259238	0.093669826	1.891645605	*0.043315005*	2.369792082	0.366558893
35	Valine	Negative Hilic	1.753550671	0.174813102	2.384865691	0.113296857	61.62403609	*0.040640995*
36	Phenylalanine	Negative Hilic	0.935194029	0.86993207	0.409014557	*0.042444283*	0.791986697	0.090863383
37	Tryptophan	Negative Hilic	0.371330134	0.081424087	0.192753035	*0.042524748*	0.01204981	*0.032135949*
38	4-Hydroxybenzaldehyde	Positive RP	0.038566578	0.338542448	0.08710944	*0.030486282*	2.748006594	0.55634682
39	2-Quinolinecarboxylic acid	Positive RP	1.284244421	0.178306336	1.296689552	*0.039303244*	1.799999525	0.963141641
40	4-Guanidinobutanoate	Positive RP	0.542825386	0.391616229	1.237852105	0.544874186	1.46736347	0.207158492
41	4-Pyridoxate	Positive RP	0.906943765	0.438937202	0.87509599	0.296192773	0.067630149	0.857936761
42	Creatinine	Positive RP	1.253400463	0.16561792	1.26221635	0.080361469	2.527037031	0.902938632
43	Deoxycarnitine	Positive RP	1.26442431	0.070202975	2.712227268	0.414190457	0.165367546	0.531546157
44	d-pantothenic acid	Positive RP	2.54453839	*0.010420678*	1.630641604	*0.003710638*	1.16289204	0.095092403
45	Guanosine	Positive RP	1.145849982	0.355689984	1.129363317	0.50751644	5.768618975	0.89840141
46	Hippurate	Positive RP	1.127259102	0.136289877	1.406303815	*0.016533704*	0.219676668	0.131795401
47	Hypoxanthine	Positive RP	1.359667365	0.457112959	1.608746416	0.099129029	7.05463603	0.809897126
48	Carnitine	Positive RP	1.90728994	0.143435009	1.564029523	0.172039547	0.018010572	0.589277748
49	Proline	Positive RP	1.1825476	0.10029429	1.364221698	*0.019455593*	1.09422421	0.087632586
50	*N*-Acetyl-d-Tryptophan	Positive RP	0.513159962	0.129235482	0.496551741	*0.022394724*	0.019329765	0.799179294
51	Nicotinamide	Positive RP	1.854686932	0.213557125	3.014205827	0.199623561	294.7130137	0.171017445
52	Phenethylamine	Positive RP	0.947278292	0.830253378	1.539430825	0.158597412	0.158329201	0.080856795
53	Pipecolate	Positive RP	1.25016381	0.293213253	1.427971372	0.207201663	1.071570022	0.09885495
54	Pyridoxal	Positive RP	1.217768919	*0.031096573*	1.27808282	0.111845597	0.112332187	0.515255364
55	Pyridoxine	Positive RP	1.087248254	0.317809478	1.096173651	0.700340773	4.820930313	0.965070128
56	Cytidine	Positive RP	2.322579759	0.180942623	2.507571618	0.258966415	29.54734542	0.869145933
57	Citrulline	Positive RP	1.24280421	0.682704165	0.603931311	0.531030346	0.000524472	0.53161591
58	Inosine	Positive RP	1.899386369	0.182257102	1.390470066	0.310097325	50.39782881	0.198914094
59	Cytosine	Positive RP	2.198331093	0.138106621	2.197735973	0.21587362	0.096448908	0.999444736
60	Uracil	Positive RP	0.944308633	0.227098922	1.026581624	0.238988701	0.184148495	0.131452792
61	Guanine	Positive RP	1.262679439	0.538371956	1.28191787	0.421569109	97.45431759	0.983003715
62	Folate	Positive RP	1.330385823	0.627752558	0.447595415	0.203196636	0.015697847	0.219888549
63	Betaine	Positive RP	2.045353528	0.181453617	2.995576467	0.1168387	2.012165194	*0.007590226*
64	Pyroglutamate	Positive RP	0.755668365	0.123082312	0.807394884	0.226135842	1566.855327	0.704018417
65	Adenosine	Positive RP	1.371651672	0.378510887	0.894426306	0.530775604	41.39432776	*0.044975022*
66	2-Hydroxyphenylacetate	Positive RP	1.100243522	0.374777295	1.16996763	0.295039165	0.890312864	0.493264835
67	Lipoamide	Positive RP	1.23055559	0.104304526	1.21925258	0.072138847	0.442513586	0.929302085
68	Suberate	Positive RP	0.959311235	0.696818973	0.964122724	0.863292691	5.735613668	0.985533593
69	4-Methylcatechol	Positive RP	0.816546234	0.152010857	0.621372018	*0.037113323*	0.807864486	*0.0491021*
70	Uridine	Negative RP	1.337277003	0.490020692	1.13709077	0.724444642	5.622283342	0.382538807
71	4-Acetamidobutanoate	Negative RP	4.059047216	0.120056303	5.167043856	0.096549799	0.000822875	0.158797722

Italics values represent metabolites with significant values *p* < 0.05.

### Significant changes in metabolites during myogenic progression

**Figure 2:**
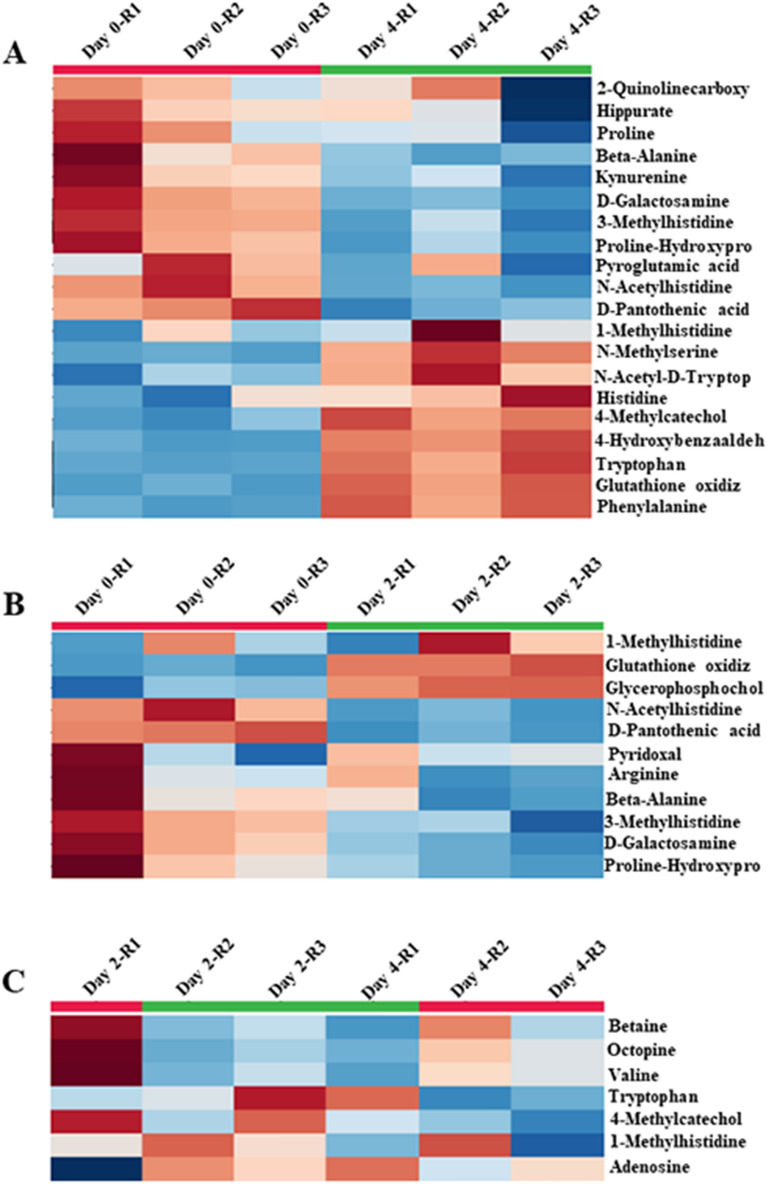
Heat map displaying metabolites that were significantly altered over the progression of myogenic culture. (**A**) 20 metabolites that were differentially altered from proliferating myogenic culture (Day 0) to late differentiating culture (Day 4). (**B**) 11 metabolites that were differentially altered from proliferating (Day 0) to early differentiating myogenic culture (Day 2). (**C**) 7 metabolites that were differentially altered from early (Day 2) to late differentiating culture (Day 4).

Using the MetaboAnalyst 4.0 software package, a total of 23 unique metabolites were identified that displayed significantly altered levels between proliferating and differentiation cultures, including 8 common between early and late differentiating cultures (Fig. 2A,B). Of these, 20 metabolites were observed to be significantly altered between proliferating and late differentiating cultures (Day 0 and Day 4) and 11 metabolites between proliferating and early differentiating cultures (Day 0 and Day 2), suggesting a global shift in metabolism when human myogenic cell progress from proliferation to a terminally differentiated state. Expectedly, numbers of metabolites showing differential expression between early and late differentiating cultures were smaller with 7 metabolites displaying significant differences in the levels of Betaine, Octopine, Valine, Tryptophan, 4-Methylcatechol, 1 Methylhistidine, and adenosine (Fig. 2C). Table 1 lists the changes in ratios of metabolites from proliferating to early differentiation (Day 0 to Day 2), early to late differentiation (Day 2 to Day 4), and from proliferation to late differentiation (Day 0 to Day 4).

**Figure 3:**
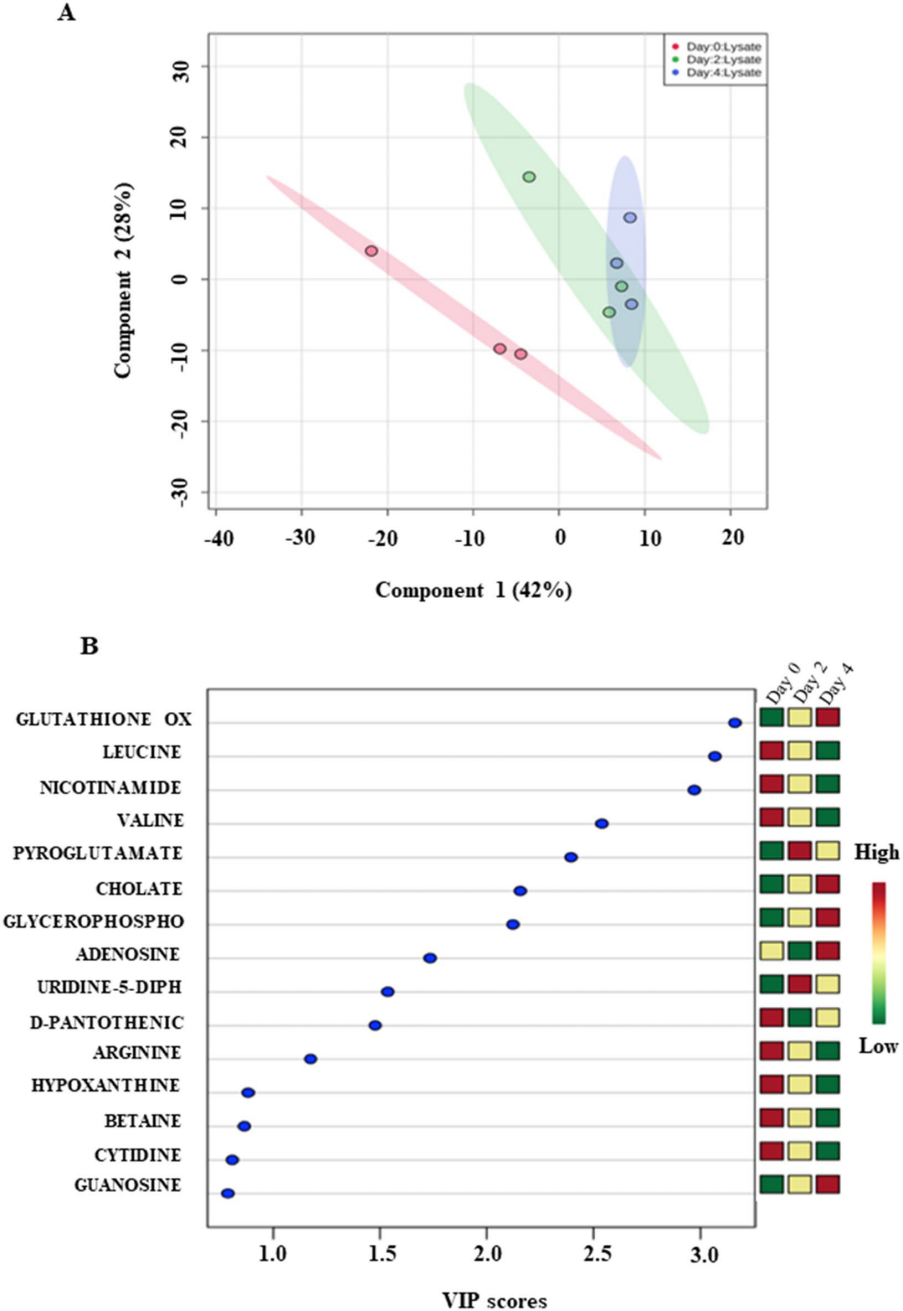
(**A**) Partial least square discriminate analysis (PLS-DA) scores plot of proliferating (Day 0), early differentiating (Day 2) and late differentiating myogenic culture (Day 4). First component versus second component showing the separation of myogenic culture (Day 0) to early (Day 2) and late differentiating myogenic culture (Day 4). Component 1 and 2 explaining about 70% of the data. (**B**) VIP scores with the corresponding expression heat map. Green and red indicates the low and high metabolites levels respectively.

To identify those metabolites with the most important features that contributed to differences between the 3 groups, we performed a PLS-DA plot (Fig. 3A). The PLS-DA score plot with component one accounting for 42% of the variation and component two explaining 28% of the variation, shows a clear separation between proliferation and differentiation, as well as a distinction between early and late differentiation (Fig. 3A). The PLS-DA analysis showed a distinct separation (R2Y = 0.9) and good predictability (Q2 = 0.56) with an accuracy of 45% (Supplementary Fig. 1C). The variable importance in projection (VIP) score plot was used to identify the top 11 most important metabolite features between the 3 different groups with scores greater than 1 and that contributed to the classification (Fig. 3B). In order to confirm that the variation in concentration of the identified metabolites was statistically significant, a *t* test was done to identify the important discriminants with a threshold of *p* < 0.05 (Table 1). We identified 6 such significant metabolites including Glutathione oxidized, valine, Glycerophosphocholine, Adenosine, d-pantothenic acid, and Arginine (Fig. 3B, Table 1). Of these metabolites, d-pantothenic acid, Arginine, and Valine were significantly upregulated in proliferating myoblasts, while, Glutathione oxidized, Glycerophosphocholine, and Adenosine were upregulated in late differentiating cultures (Fig. 3B).

### Metabolomic pathway analysis in myogenic progression

**Figure 4:**
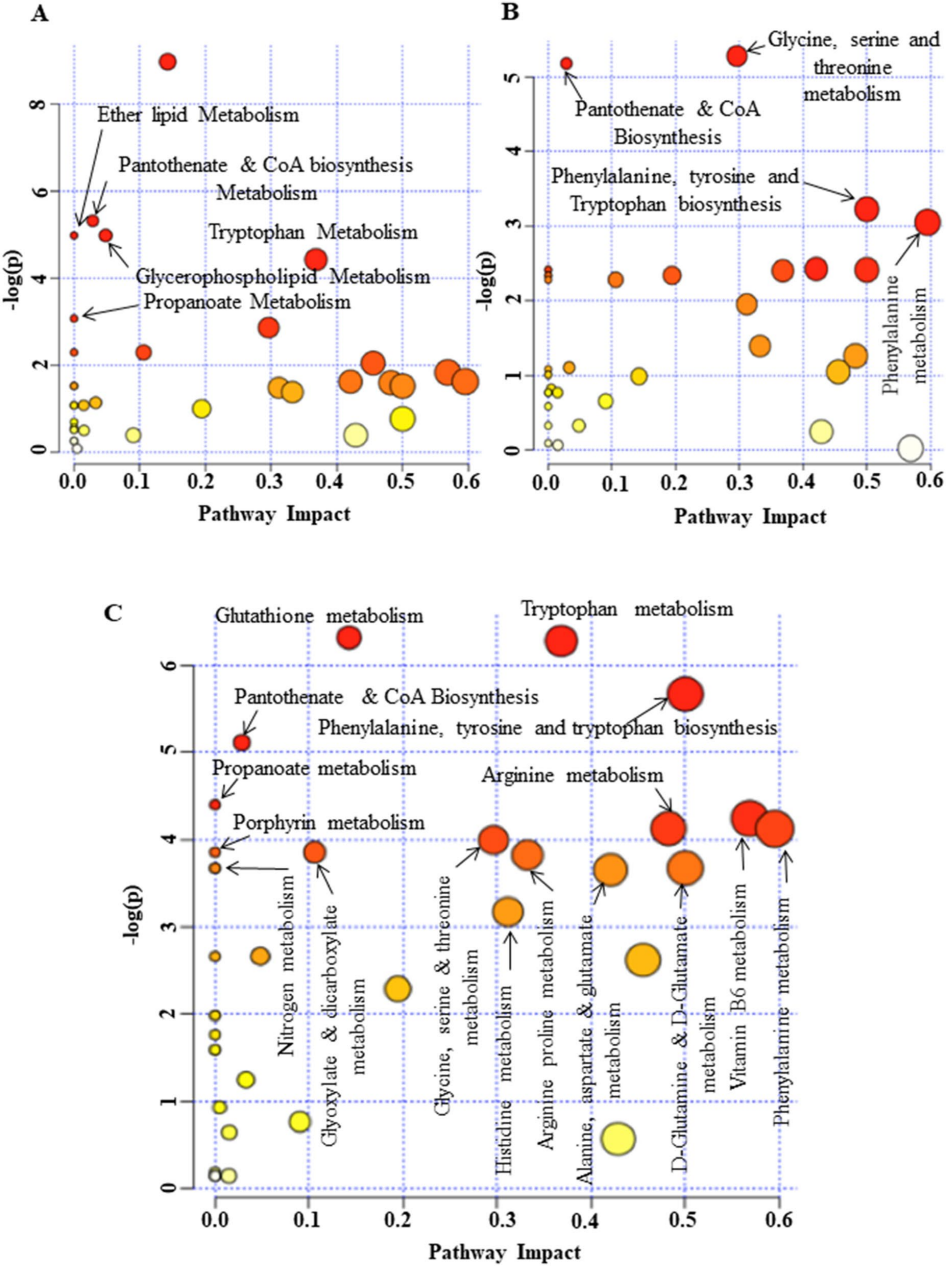
Summary of pathway analysis with Ingenuity pathway analysis based on KEGG database. All the significantly match (*p* value < 0.05) were labeled. The color and size of each circle was based on *p* value and pathway impact value, respectively. Pathways that were significantly altered between (**A**) proliferating myoblasts (Day 0) and early differentiating culture (Day 2), (**B**) proliferating (Day 0) and late differentiating culture (Day 4), and (**C**) early and late differentiating cultures.

**Figure 5:**
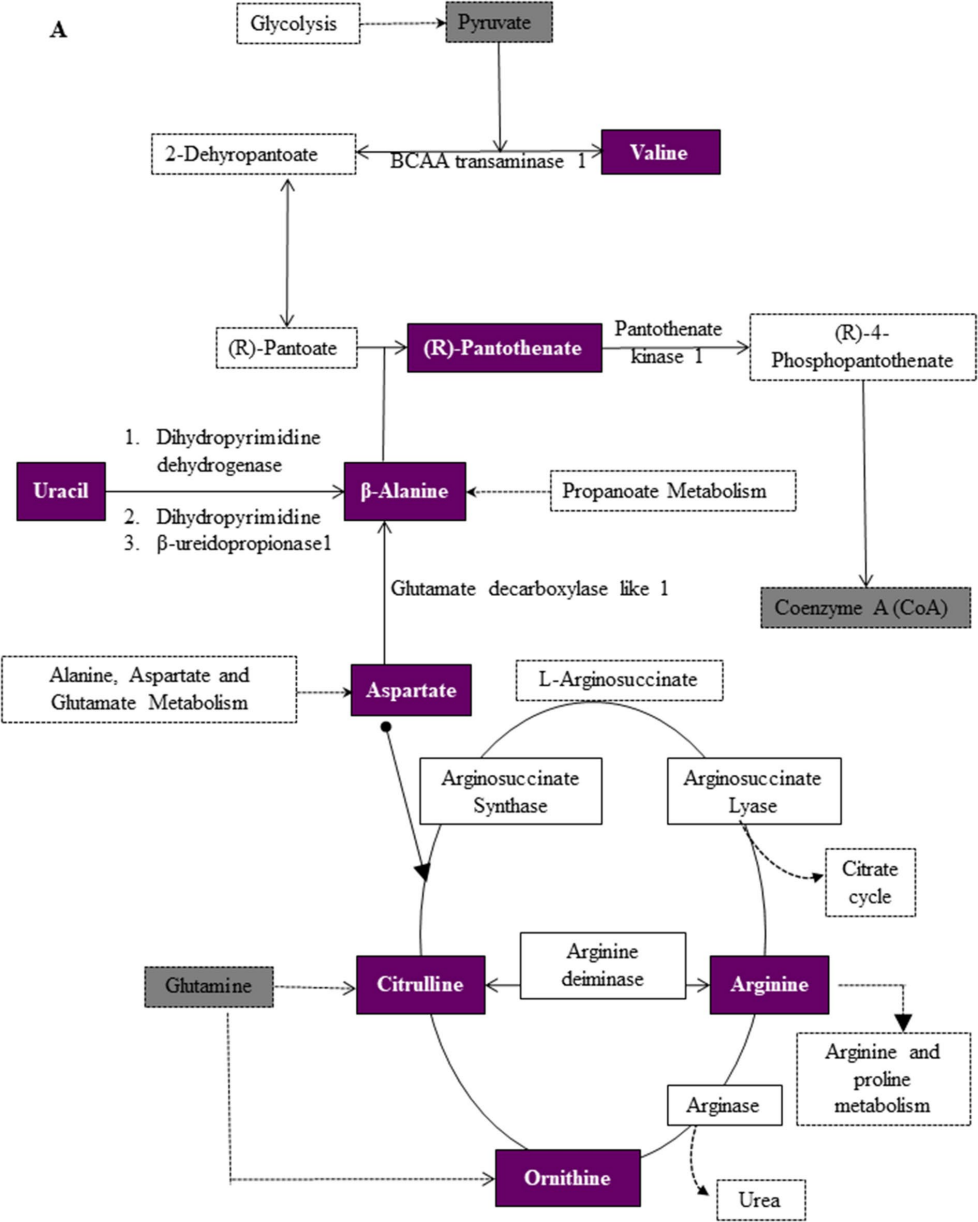

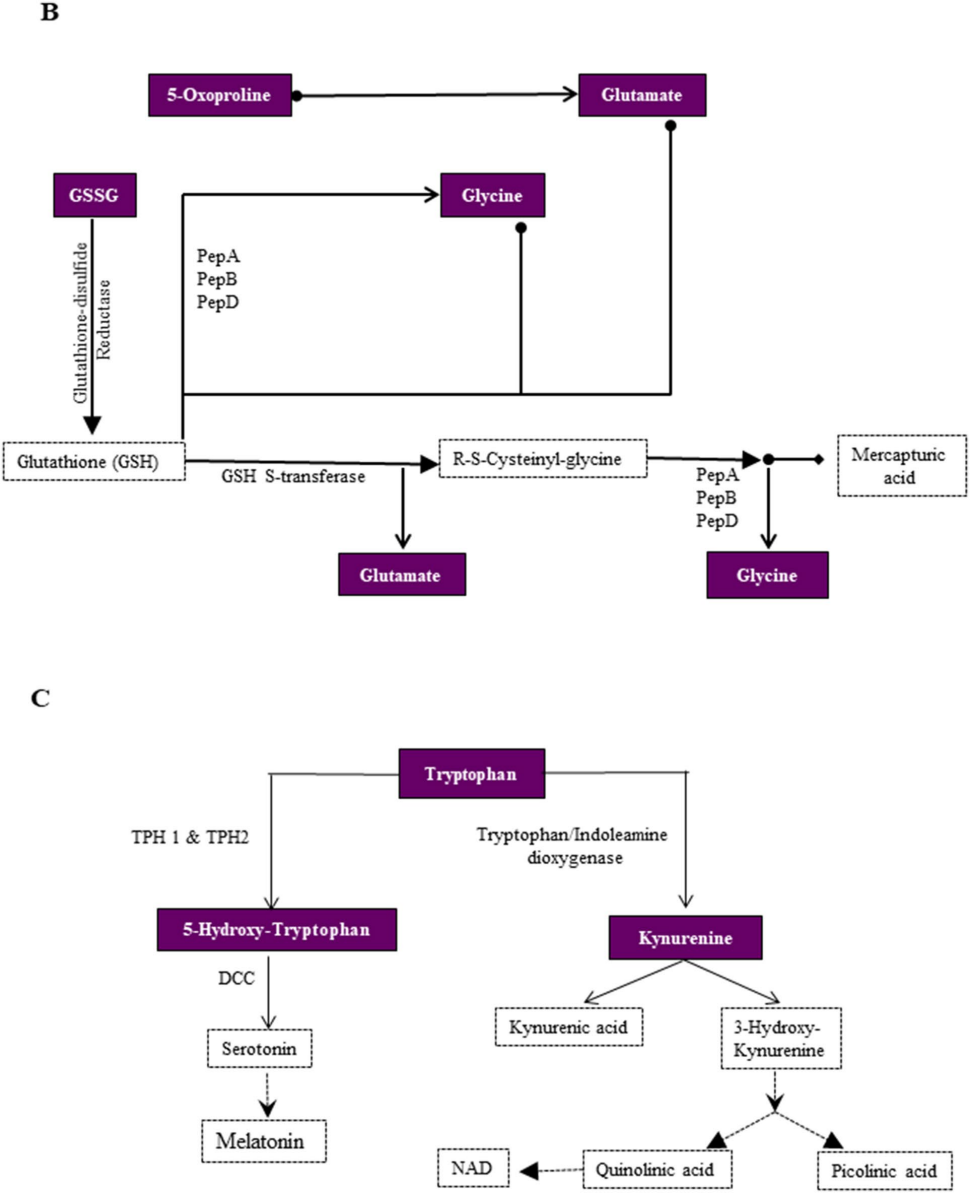
Representative metabolic pathway maps of significantly altered metabolites during proliferation and differentiation. Key metabolites related to (**A**) Pantothenate and CoA biosynthesis and Arginine and Proline metabolism that display significant alteration during proliferation and (**B**) Glutathione metabolism, (**C**) Tryptophan metabolism during differentiation. Colored boxes represent identified metabolites. Images were modified from map00770 and map00220 respectively for (**A**), map00480 for (**B**), and map00380 for (**C**) from the KEGG database (https://www.kegg.jp).

To investigate which of the metabolic pathways were significantly altered during myogenic progression, we performed Pathways analysis with MetaboAnalyst 4.0 and identified the Pantothenate metabolic pathway and Coenzyme A (CoA) biosynthesis to be significantly regulated throughout myogenic progression (Fig. 4, Table 2). Specifically, Pantothenate and CoA biosynthesis was upregulated in proliferating cultures compared to differentiating myogenic cultures (Fig. 4, Table 2). Additionally, we detected several metabolites indicating a dominant role for Arginine and Proline metabolism in proliferating myoblasts (Fig. 4, Tables 1, 2). Metabolic pathway maps displayed key metabolites related to the Pantothenate and CoA biosynthesis pathway that were altered during proliferation including Pantothenate, β-alanine, Uracil, Valine, and Aspartate, while those related to Arginine and Proline metabolism included Arginine, Ornithine, Citrulline and Aspartate (Fig. 5A).

**Table 2:** Metabolic pathways displaying significant changes in the primary human myoblasts during the course of myogenic progression.

S. no	Pathways displayed significant changes during Day 0 to Day 2 transformation	Match status	*p* value	FDR
1	Pantothenate and CoA Biosynthesis	5/19	0.0049	0.062407
2	Glycerophospholipid Metabolism	1/36	0.0069	0.062407
3	Ether lipid metabolism	1/20	0.0069	0.062407
4	Tryptophan Metabolism	3/41	0.0120	0.086932
5	Pantothenate Metabolism	1/23	0.0466	0.27999
**Pathways displayed significant changes during Day 2 to Day 4) transformation**				
1	Glycine, Serine and Threonine Metabolism	2/33	0.0051	0.10159
2	Pantothenate and CoA Biosynthesis	5/19	0.0056	0.10159
3	Phenylalanine, Tyrosine and Tryptophan Biosynthesis	1/4	0.0396	0.26212
4	Phenylalanine Metabolism	4/10	0.0472	0.26212
**Pathways displayed significant changes during Day 0 to Day 4 transformation**				
1	Glutathione metabolism	5/28	0.0018	0.033886
2	Tryptophan Metabolism	3/41	0.0018	0.033886
3	Phenylalanine, Tyrosine and Tryptophan Biosynthesis	1/4	0.0034	0.041434
4	Pantothenate and CoA Biosynthesis	5/19	0.0060	0.054196
5	Pantothenate Metabolism	1/23	0.0122	0.058348
6	Vitamin B6 Metabolism	3/9	0.0143	0.058348
7	Arginine Biosynthesis	5/14	0.0161	0.058348
8	Phenylalanine Metabolism	4/10	0.0161	0.058348
9	Glycine, Serine and Threonine Metabolism	2/33	0.0184	0.058348
10	Glyoxylate and Dicarboxylate Metabolism	2/32	0.0211	0.058348
11	Porphyrin Metabolism	2/30	0.0211	0.058348
12	Arginine and Proline Metabolism	6/38	0.0211	0.058348
13	d-Glutamine and d-Glutamate Metabolism	1/6	0.0254	0.058348
14	Butanoate Metabolism	1/15	0.0254	0.058348
15	Nitrogen Metabolism	1/6	0.0254	0.058348
16	Alanine, Aspartate and Glutamate Metabolism	2/28	0.0259	0.058348
17	Histidine Metabolism	5/16	0.0419	0.088755

Of the 12 unique pathways upregulated during differentiation, vitamin B6, Glyoxylate and Dicarboxylate, Nitrogen, Glutathione, and Tryptophan metabolism were upregulated in late differentiation (Table 2). Pathway analyses between early and late differentiating cultures identified significant alterations primarily in specific amino acid metabolism such as Phenylalanine, Tyrosine, Glycine, Serine and Threonine metabolism (Fig. 4, Table 2). Metabolic pathway maps showed key metabolites related to the Glutathione pathway were altered during differentiation including Glutathione oxidised (GSSG), Glycine, Glutamate, and 5-Oxoproline, while metabolites related to the Tryptophan metabolism pathway were Kynurenine and 5-Hydroxy tryptophan (Fig. 5B,C).

## Discussion

Skeletal muscle performance in the context of physical activity and endocrine regulation of other organ systems is critically dependent on energy demands of individual myogenic cells. These cells are characterised by specific metabolite profiles that signify as biomarkers of a particular stage of myogenic progression^13,14^. While gene expression and in vivo functional studies have indicated the importance of specific factors for muscle mass maintenance and physiology, less is known about measureable changes in metabolites corresponding to these pathways, the tissue source, and the temporal kinetics of these metabolites during the course of myogenic progression^16–18^. Using two multivariate statistical methods of PCA and PLS-DA analyses, our results identify the predominant metabolites and pathways important for proliferation and distinct differentiation stages during myogenic progression in human primary myoblasts.

From our results we found that a key pathway to be active in proliferation was Arginine and Proline metabolism with Arginine displaying a VIP score > 1 (Fig. 3B). Specifically, we found that proline–hydroxyproline was upregulated 1.7-fold in proliferating myoblasts over early differentiating cultures and 2-fold over later differentiating cultures (Fig. 3A). Both proline and hydroxyproline comprise 30% of the amino acids in collagen, a vital component in the extracellular matrix of skeletal muscle^19^. These amino acids contribute to the stability of the helices in collagen and are responsible for the stiffness of this polymer indicating that these are necessary components in creating bio-artificial muscles in tissue engineering^19,20^. The increase in metabolite levels of proline and hydroxyproline in the early stages suggest a preparatory step in myogenic progenitors for terminal differentiation. It has also been reported that metabolomic analysis of aged muscles has revealed a reduction in proline and hydroxyproline that might be responsible for the progressive loss of muscle mass and function in sarcopenia, in accordance with dysfunctional satellite cell activation in aging muscles^11^.

Our analysis revealed that one of the pathways dominating the proliferative phase is the Pantothenate metabolic pathway and CoA biosynthesis with d-pantothenic acid displaying a high VIP score (Fig. 3B). Pantothenic acid is the primary substrate for Pantothenate kinase, the rate limiting step in CoA biosynthesis, accounting for about 66% of CoA in skeletal muscle, the highest amount than in any other tissue^21–23^. In muscle energy metabolism, CoA contributes to the fatty acyl-CoA pool in mitochondria via the carnitine shuttle system, that is subsequently used for the carnitine palmitoyl-transferase 2 (CPT2) mediated transesterification of acyl-carnitine to carnitine and acyl CoA, for mitochondrial fatty acid β-oxidation, oxidative decarboxylation of pyruvate to acetyl CoA, and finally as a key substrate for α-ketoglutarate in the TCA cycle^24–27^. Importantly, CoA functions as a substrate for the enzyme, acyl CoA synthetase (ACS) that enables a critical fuel selection switch from glucose to fatty acids (FA) during activity^28^. Indeed, impaired ACS function result in a significant decline in FA oxidation, heightened insulin sensitivity, depleted glucose reserves resulting in a form of hypoglycaemia that could not be compensated by hepatic gluconeogenesis, and reduction in muscle endurance capacity^28^. Physiological regulation of CoA became evident in a study examining the effects of altering CoA levels in skeletal muscle that resulted in altered mitochondrial morphology, lowered ATP levels, increased oxidative stress, reduced grip strength and endurance in exercise^29^ Thus, it may seem that Pantothenate and CoA profiles and their metabolites in muscle cells might potentially be altered during specific pathological situations.

Another key metabolite that was modestly, but significantly upregulated in differentiating human myoblasts was Pyridoxal, suggesting the importance of vitamin B6 and one carbon metabolism (OCM) in myogenic progression. Specifically, vitamin B6 is a source for pyridoxal phosphate (PLP), a coenzyme required for several phases of OCM, further highlighting the importance of this dietary nutrient in aiding normal muscle growth and function, especially during muscle development and postnatal regeneration. Additionally, recent reports suggest that PLP is essential for the synthesis of carnosine, a histidine containing dipeptide that is prevalent in Type ll glycolytic fibers, with increased PLP levels present in highly contractile muscle, suggesting a role for vitamin B6 metabolism in muscle regeneration^30,31^.

Of the metabolites present at increasing levels during differentiation, we found glutathione metabolism particularly GSSG displaying the highest VIP score of 3.2, to be significantly upregulated more than 2-fold above proliferating myoblasts, indicating the importance of regulation of oxidative stress in myogenic progression (Fig. 3B). These results are concordant with a recent report examining antioxidant transcription and myogenic differentiation in satellite cells, indicating that a pro-oxidative environment is essential for differentiation^32^. Despite the observation that GSSG is a notoriously difficult compound to quantify by mass spectrometry, it is highly unlikely that its presence is an artefact of the methodology, since we detect this compound at retention time 8.3 with very good peak shape and ion intensity using HILIC negative mode as is indicated by high resolution EIC analysis (Supplementary Fig. 4). Increasing concentrations of tryptophan, a precursor of serotonin and IGF-1 that is associated with increasing muscle mass, suggest the direct impact that this essential amino acid has on differentiation. Indeed, it has been shown that reduced levels of tryptophan are associated with sarcopenia, while its metabolite kynurenine has been observed to accumulate in the peripheral tissues of rats with advanced age^33,34^. Specifically, mice fed with tryptophan, but not its metabolite kynurenine showed increased expression of myogenic, as well Akt1 and Akt2, two factors essential for myogenic differentiation and myotube maturation^35^. This suggests that the increase in muscle mass on tryptophan administration arises because of its requirement during terminal myogenic differentiation.

### Conclusion

Overall, our study aims to use high throughput metabolomics analysis as a foundational study to characterise skeletal muscle-specific metabolic changes during myogenic progression that includes proliferating, early differentiating and late differentiating human myogenic cultures. Using an untargeted metabolomics approach coupled to multivariate analyses, this is the first study to implicate specific pathways such as Pantothenate metabolism and CoA biosynthesis and arginine and proline metabolism during the proliferation step of myogenic progression. Several unique pathways including vitamin B6, Tryptophan, Glutathione, and Glyoxylate and Dicarboxylate metabolism were identified as the key pathways that are altered during the differentiation phase of myogenic progression. Although the study captures a wide unbiased landscape of metabolites, this could be a precursor for future studies pursuing individual metabolites that are perturbed during myogenic differentiation for specific muscle diseases.

## Materials and methods

### Cell culture

Human skeletal muscle myoblasts (hSKmc) (CC-2580), growth media, supplements, and serum (CC-3245) were purchased from Lonza (Walkersville, MD). HSMM differentiation medium (DM) was prepared by adding 2% horse serum to DMEM (Hyclone). HSMM were maintained in growth media for 3–4 days till cultures reached 70% confluence followed by three washes in phosphate buffered saline (PBS, pH 7) and switched to DM. Intracellular metabolites from cell lysates were collected from Day 0 cultures (before addition of DM), Day 2 and Day 4 cultures (DM added for 2 and 4 days respectively). For kinetic analysis of intracellular metabolites, hSKmc cultures for Day 0, 2, and 4 underwent three washes in PBS, scraped in RIPA lysis and extraction buffer (Cat # 89900, ThermoFisher Scientific), and stored at – 80 °C.

### Sample preparation

Metabolites were extracted from the quenched cells using 100% methanol extraction method. Cells pellet frozen at − 80 °C was thawed for 10 min and resuspended in 500 μl of 100% chilled methanol (MS-grade, Sigma) and vortexed for 1 min and frozen at − 80 °C for 10 min. The freeze–thaw cycle was repeated twice followed by pelleting of cells by centrifugation at 15,000*g* for 10 min at 4 °C. After centrifugation each supernatant (~ 400 μl) was collected in a separate microfuge tube without disturbing the pellet. Each fraction (100 μl) was dried using a speed vacuum at room temperature for 20–25 min. Samples were stored at − 80 °C till further analysis. For sample injection, each sample was resuspended in 25 μl of methanol water mixture (3:17, methanol: water), vortexed briefly and centrifuged at 11,000 rpm for 10 min at 4 °C.

### Measurement of metabolites

Orbitrap Fusion mass spectrometer (Thermo Scientific) coupled with heated electrospray ion source was used for data acquisition. Data acquisition methods have been followed as per published protocols^36,37^ with minor modifications. Briefly for MS1 mode, mass resolution was kept at 120,000 and for MS2 acquisition, mass resolution was 30,000. Mass range of data acquisition was 60–900 da. Extracted metabolites were separated on UPLC ultimate 3,000. Data were acquired on reverse phase and HILIC column and positive and negative ionization mode both. Reverse phase column was HSS T3 and HILIC column was XBridge BEH Amide (Waters Corporation). For polar compound separation, solvent A was 20 mM ammonium acetate in the water of PH 9.0 and mobile phase B was 100% acetonitrile. The elution gradient starts from 85% B to 10% B over 14 min with flow rate of 0.35 ml/min. For reverse phase, Solvent A was water and B was methanol with 0.1% formic acids added in both. The elution gradient starts with 1% B to 95% B over 10 min with flow rate 0.3 ml/min. sample injection volume was 5ul. Pool quality control (QC) sample was run after every five samples to monitor signal variation and drift in mass error. Data matrices have been provided in Supplementary Table 1 and representative spectra of one of the samples acquired in positive and negative modes of HILIC and RP have been provided in Supplementary Fig. 3.

### Data processing

All LC/MS acquired data has been processed using the Progenesis QI for metabolomics (Water Corporation) software using the default setting. The untargeted workflow of Progenesis QI was used to perform retention time alignment, feature detection, deconvolution, and elemental composition prediction. Metascope plug of Progenesis QI has been used for the in-house library with accurate mass, fragmentation pattern and retention time for database search. We have also used online available spectral library for further confirmation of identification. Cut-off for retention time match was 0.5 min and spectral similarity was more than 30% fragmentation match in Progenesis QI. Peaks that had a coefficient of variation (CV) less than 30% in pool QC samples were kept for the further analysis of data. Additionally, manual verification of each detected feature has been done for the selection of right peaks.

### Statistical analysis

For each of the stages of myogenic progression (Day 0, 2, and 4), three independent experiments with equal number of cells counts were performed. Processing of the raw data lead to identification of total 71 metabolites at all the three stages (Day 0, Day 2 and Day 4) of myogenic progression. All the further statistical and functional analysis including the PCA, heat map, molecular pathways identification, analysis of variance (ANOVA) was done based on the identified peaks intensity using the online freely available Metaboanalyst 4.0 software. For analysis of PCA, heatmaps, ANOVA data were put in matrix, with samples in rows and features in columns. Before final analysis data integrity check was performed and raw data were normalized by sum methods and scaling was done using the Pareto scaling.

## Supplementary information

Supplementary information.

## Data Availability

Data deposited to MetaboLights with ID-MTBLS1818 (https://www.ebi.ac.uk/metabolights/MTBLS1818).

## References

[1] Lepper C, Partridge TA, Fan CM (2011). An absolute requirement for Pax7-positive satellite cells in acute injury-induced skeletal muscle regeneration. Development.

[2] Murphy MM, Lawson JA, Mathew SJ, Hutcheson DA, Kardon G (2011). Satellite cells, connective tissue fibroblasts and their interactions are crucial for muscle regeneration. Development.

[3] Sambasivan R (2011). Pax7-expressing satellite cells are indispensable for adult skeletal muscle regeneration. Development.

[4] Schultz E (1996). Satellite cell proliferative compartments in growing skeletal muscles. Dev. Biol..

[5] Biressi S, Gopinath SD (2015). The quasi-parallel lives of satellite cells and atrophying muscle. Front. Aging Neurosci..

[6] Clish CB (2015). Metabolomics: an emerging but powerful tool for precision medicine. Cold Spring Harb. Mol. Case Stud..

[7] Matsuda R, Uchitomi R, Oyabu M, Hatazawa Y, Kamei Y (2019). Metabolomic analysis of C2C12 myoblasts induced by the transcription factor FOXO1. FEBS Lett..

[8] Meinke P, Limmer S, Hintze S, Schoser B (2019). Assessing metabolic profiles in human myoblasts from patients with late-onset Pompe disease. Ann. Transl. Med..

[9] Saoi M (2019). Characterization of the human skeletal muscle metabolome for elucidating the mechanisms of bicarbonate ingestion on strenuous interval exercise. Anal. Chem..

[10] Sato S, Parr EB, Devlin BL, Hawley JA, Sassone-Corsi P (2018). Human metabolomics reveal daily variations under nutritional challenges specific to serum and skeletal muscle. Mol. Metab..

[11] Uchitomi R (2019). Metabolomic analysis of skeletal muscle in aged mice. Sci. Rep..

[12] Joseph J, Cho DS, Doles JD (2018). Metabolomic analyses reveal extensive progenitor cell deficiencies in a mouse model of duchenne muscular dystrophy. Metabolites.

[13] Pala F (2018). Distinct metabolic states govern skeletal muscle stem cell fates during prenatal and postnatal myogenesis. J. Cell Sci..

[14] Ryall JG (2017). Simultaneous measurement of mitochondrial and glycolytic activity in quiescent muscle stem cells. Methods Mol. Biol..

[15] Seldin MM (2018). A strategy for discovery of endocrine interactions with application to whole-body metabolism. Cell Metab..

[16] Cohen S, Nathan JA, Goldberg AL (2015). Muscle wasting in disease: molecular mechanisms and promising therapies. Nat. Rev. Drug Discov..

[17] Glass DJ (2005). Skeletal muscle hypertrophy and atrophy signaling pathways. Int. J. Biochem. Cell. Biol..

[18] Sakuma K, Aoi W, Yamaguchi A (2014). The intriguing regulators of muscle mass in sarcopenia and muscular dystrophy. Front. Aging Neurosci..

[19] Nelson DL, Lehninger AL, Cox MM (2005). Lehninger Principles of Biochemistry.

[20] Thorrez L, DiSano K, Shansky J, Vandenburgh H (2018). Engineering of human skeletal muscle with an autologous deposited extracellular matrix. Front. Physiol..

[21] Smith CM, Narrow CM, Kendrick ZV, Steffen C (1987). The effect of pantothenate deficiency in mice on their metabolic response to fast and exercise. Metabolism.

[22] Zano SP, Pate C, Frank M, Rock CO, Jackowski S (2015). Correction of a genetic deficiency in pantothenate kinase 1 using phosphopantothenate replacement therapy. Mol. Genet. Metab..

[23] Zhang YM, Rock CO, Jackowski S (2005). Feedback regulation of murine pantothenate kinase 3 by coenzyme A and coenzyme A thioesters. J. Biol. Chem..

[24] Bremer J, Wojtczak AB (1972). Factors controlling the rate of fatty acid -oxidation in rat liver mitochondria. Biochim. Biophys. Acta.

[25] Cooper RH, Randle PJ, Denton RM (1975). Stimulation of phosphorylation and inactivation of pyruvate dehydrogenase by physiological inhibitors of the pyruvate dehydrogenase reaction. Nature.

[26] Garland PB, Yates DW, Haddock BA (1970). Spectrophotometric studies of acyl-coenzyme A synthetases of rat liver mitochondria. Biochem. J..

[27] Oram JF, Wenger JI, Neely JR (1975). Regulation of long chain fatty acid activation in heart muscle. J. Biol. Chem..

[28] Li LO (2015). Compartmentalized acyl-CoA metabolism in skeletal muscle regulates systemic glucose homeostasis. Diabetes.

[29] Corbin DR (2017). Excess coenzyme A reduces skeletal muscle performance and strength in mice overexpressing human PANK2. Mol. Genet. Metab..

[30] Crozier PG, Cordain L, Sampson DA (1994). Exercise-induced changes in plasma vitamin B-6 concentrations do not vary with exercise intensity. Am. J. Clin. Nutr..

[31] Suidasari S (2015). Carnosine content in skeletal muscle is dependent on vitamin B6 status in rats. Front. Nutr..

[32] Rajasekaran NS, Shelar SB, Jones DP, Hoidal JR (2020). Reductive stress impairs myogenic differentiation. Redox Biol..

[33] Braidy N, Guillemin GJ, Mansour H, Chan-Ling T, Grant R (2011). Changes in kynurenine pathway metabolism in the brain, liver and kidney of aged female Wistar rats. FEBS J..

[34] Caballero B, Gleason RE, Wurtman RJ (1991). Plasma amino acid concentrations in healthy elderly men and women. Am. J. Clin. Nutr..

[35] Dukes A (2015). The aromatic amino acid tryptophan stimulates skeletal muscle IGF1/p70s6k/mTor signaling in vivo and the expression of myogenic genes in vitro. Nutrition.

[36] Choudhary E, Sharma R, Kumar Y, Agarwal N (2019). Conditional silencing by CRISPRi reveals the role of DNA gyrase in formation of drug-tolerant persister population in *Mycobacterium tuberculosis*. Front. Cell Infect. Microbiol..

[37] Naz S (2017). Development of a liquid chromatography-high resolution mass spectrometry metabolomics method with high specificity for metabolite identification using all ion fragmentation acquisition. Anal. Chem..

