# Supplementary material for: Metabolomic analysis of primary human skeletal muscle cells during myogenic progression
Source: Sci Rep. 2020 Jul 16;10:11824. doi: 10.1038/s41598-020-68796-4 (PMC7366914; doi:10.1038/s41598-020-68796-4)
Supplement: Supplementary file 1 — Supplementary information. [file 41598_2020_68796_MOESM1_ESM.pdf]

Metabolomic analysis of primary human skeletal muscle cells during myogenic progression

Ashok Kumar[1], Yashwant Kumar[1], Jayesh Kumar Sevak[1], Sonu Kumar[1], Niraj Kumar[1*], and Suchitra Devi Gopinath[1*]

Supplementary information

Supplementary Legends and Table

Supplementary Figure 1

(A) Relative mRNA levels of MyoD, Myogenin, and MyHC in proliferating hSkmcs, early and late differentiating cultures. Gene expression levels of individual genes were normalized to GAPDH. (B) PCA analysis of the metabolomics datasets from proliferating (Day 0) and differentiating cultures (Day 2 and Day 4) were performed using the MetaboAnalyst 4.0 (n=3 per sample). Plot of Day-0 (Red Pink circle) and Day-2 and Day-4 (Green and Blue) metabolites are clearly distinguished on the PC2 axis (Y-axis). (C) PLSDA cross validation plot with accuracy of around 45%, R2 90% and Q2 56%.

Supplementary Figure 2

A heat map comparing the metabolites changes during the course of differentiation of primary human myoblasts at 3 different time points (Day-0, Day-2 and Day-4). R1, R2, and R3 indicate the replicates for each sample. Distinct pattern changes between Day 0 versus Day 2 and 4 indicate a clear separation in metabolite pattern during myogenic progression from proliferation to differentiation. Red indicates that the relative contents of metabolites are high whereas blue indicates that the relative content of metabolites are low.

Supplementary Figure 3

Total ion chromatograms of one of the representative sample from HILIC, Reverse phase, positive and negative mode.

Supplementary Figure 4

EIC of GSSG with a retention time of 8.3 using HILIC negative mode

Supplementary Figure 1

A

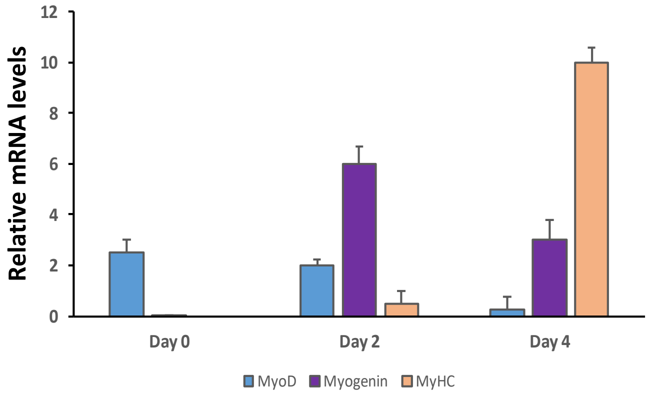

B

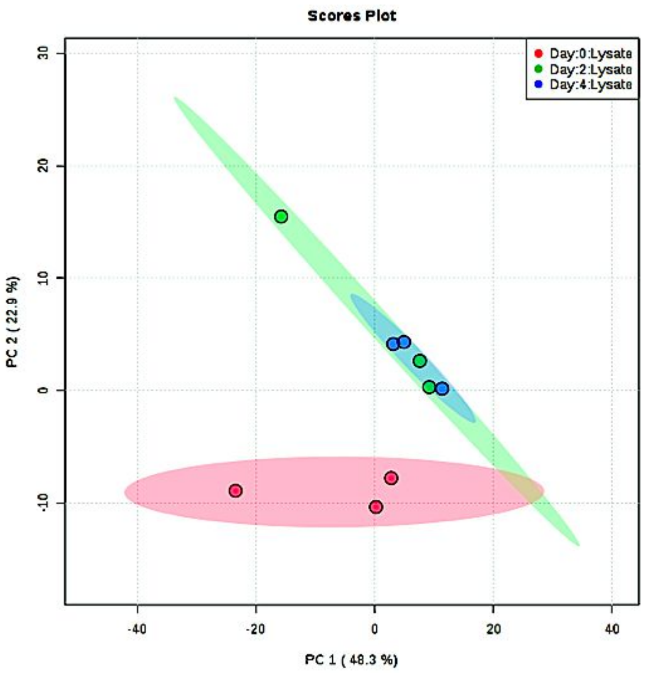

C

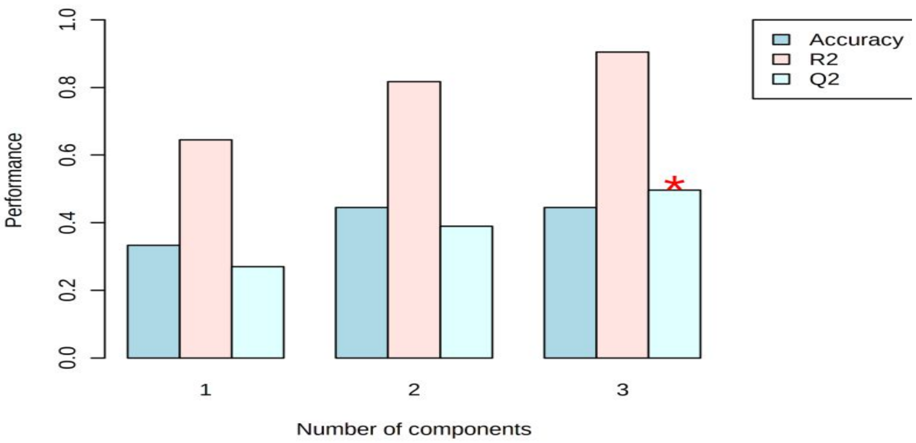

Supplementary Figure 2

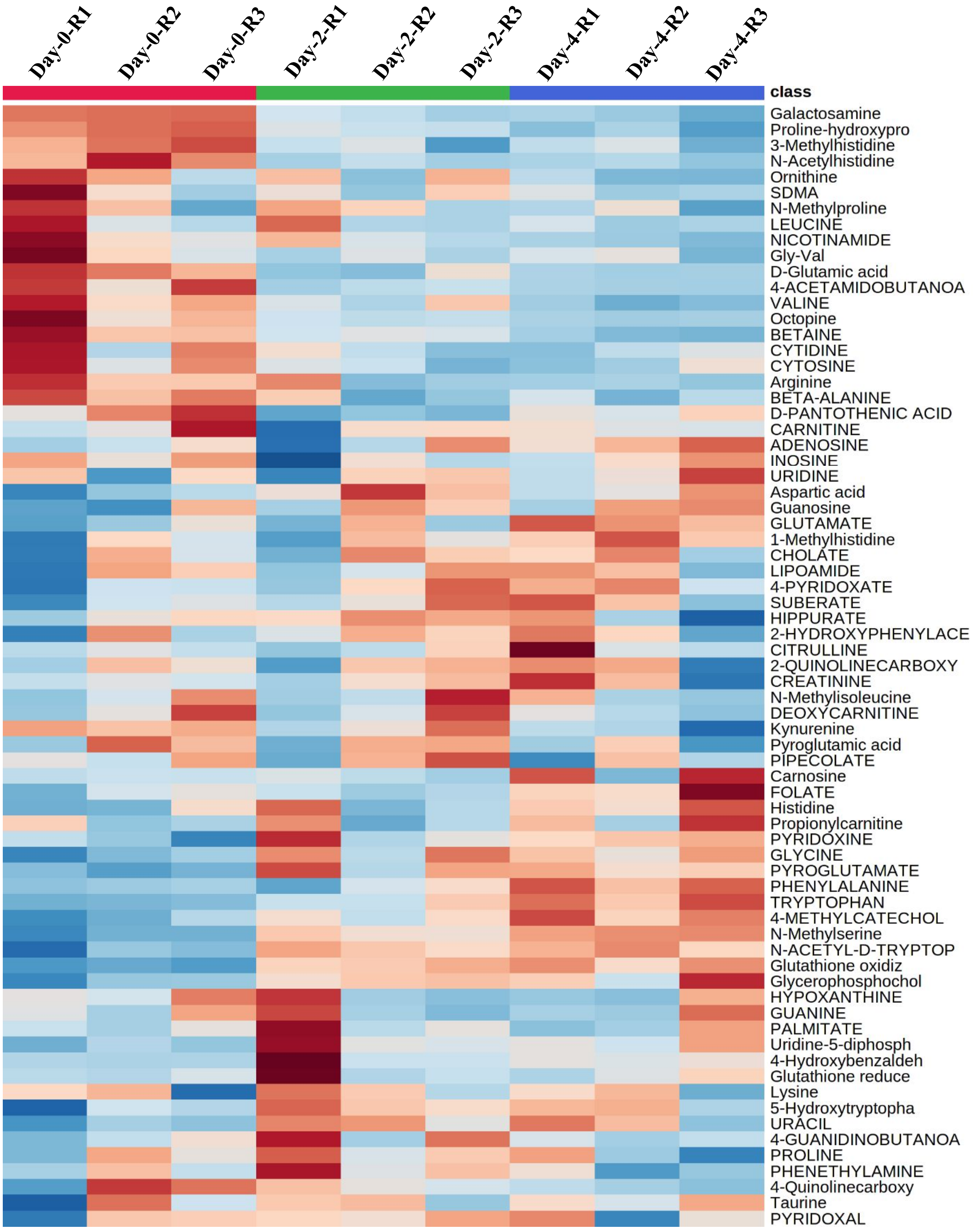

Supplementary Figure 3

HILIC_NEG_TIC

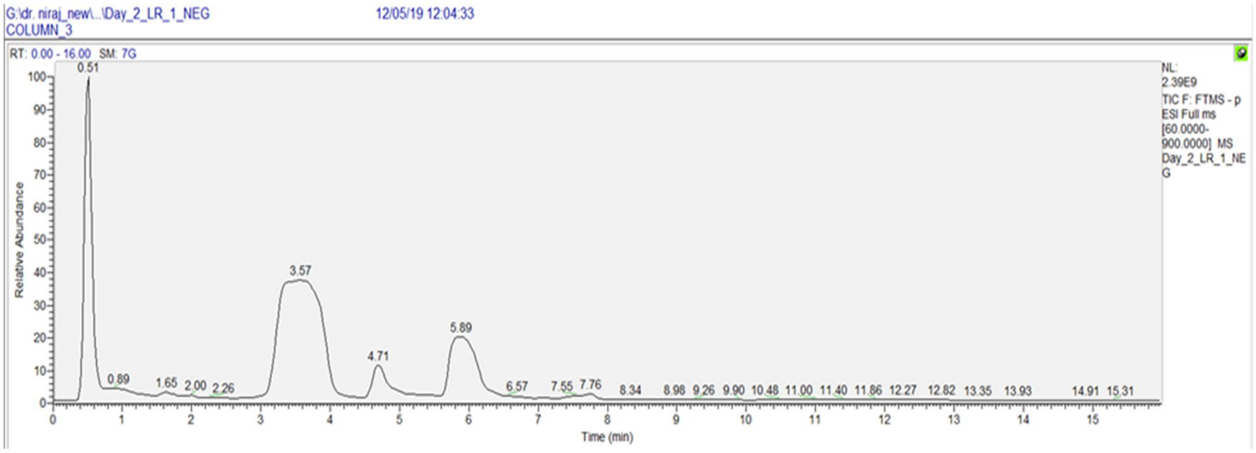

HILIC_POS_TIC

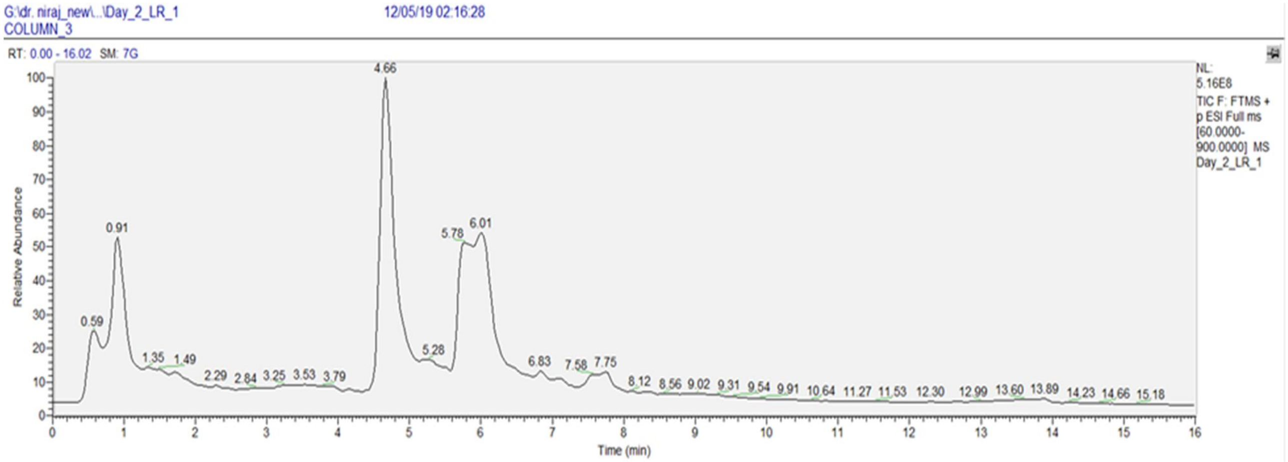

RP_NEG_TIC

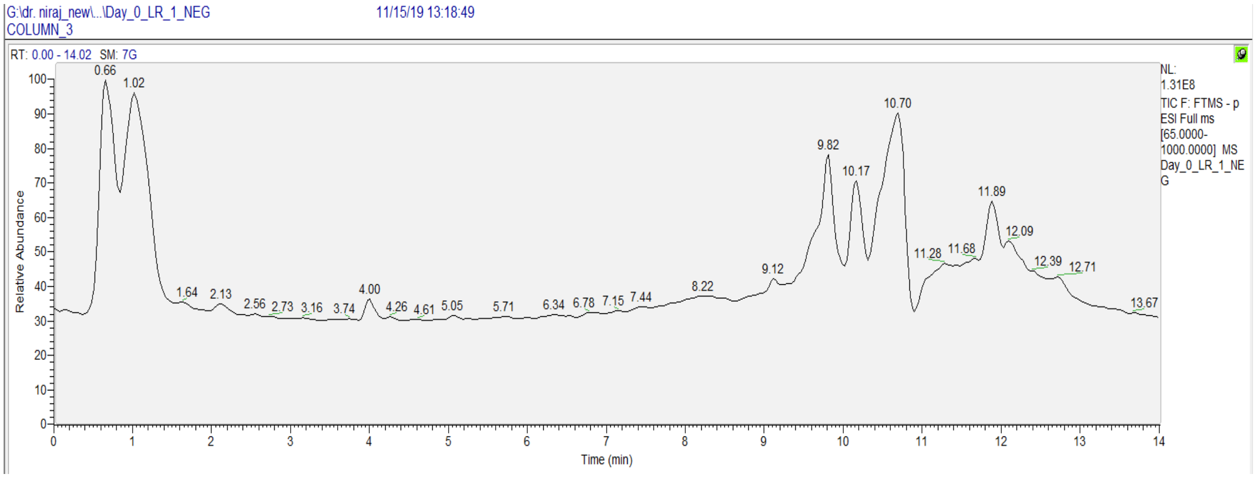

RP_POS_TIC

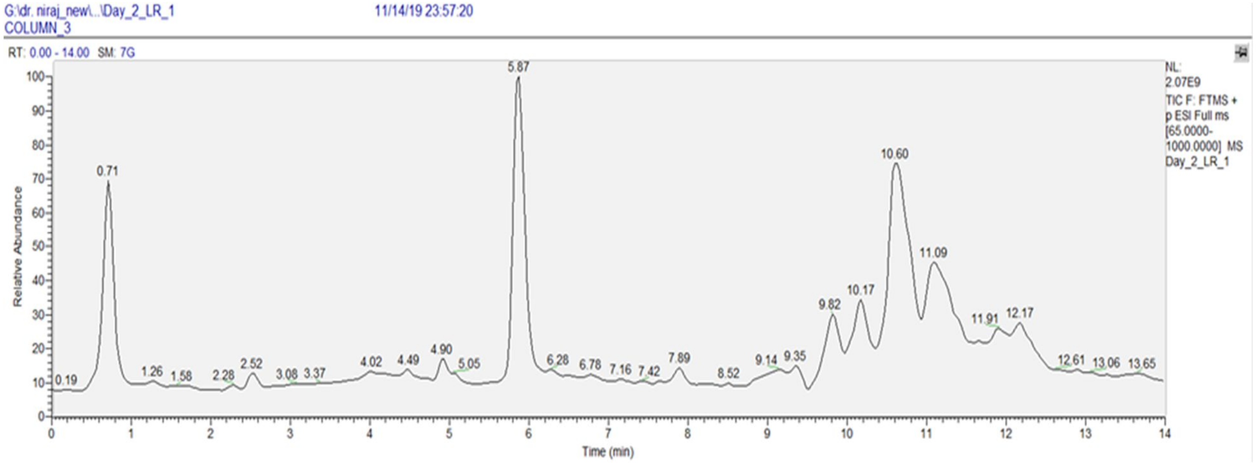

Supplementary Figure 4

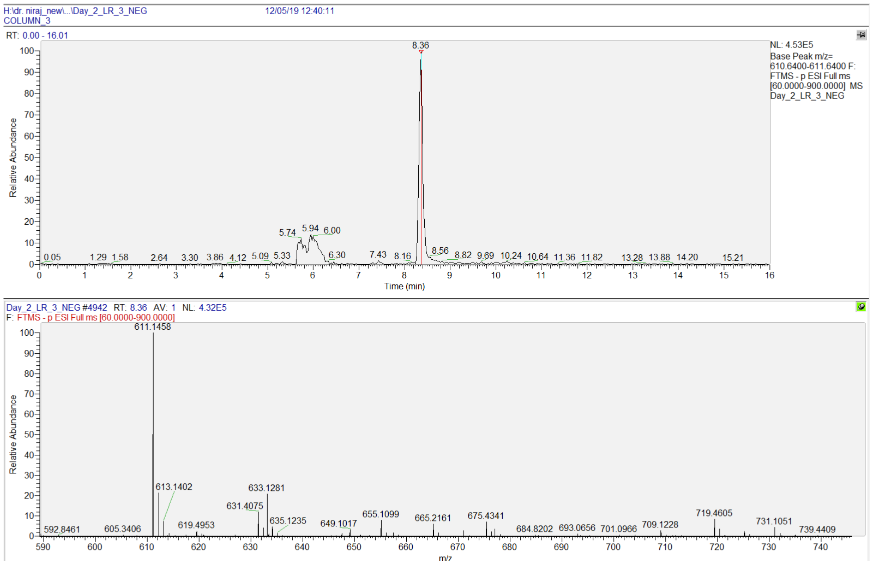

Supplementary Table-1: Metabolomics data matrices.

Accepted Description	Day_0_LR_1	Day_0_LR_2	Day_0_LR_3	Day_2_LR_1	Day_2_LR_2	Day_2_LR_3	Day_4_LR__1	Day_4_LR__2	Day_4_LR__3
Samples	Day:0:Lysate	Day:0:Lysate	Day:0:Lysate	Day:2:Lysate	Day:2:Lysate	Day: 2:Lysate	Day:4:Lysate	Day: 4:Lysate	Day: 4:Lysate
3-Methylhistidine	400.724225	333.0186895	354.8054464	212.8900124	200.3825197	142.8231979	181.1872728	195.3802023	176.0374823
4-Hydroxybenzaldehyde	18.37841648	6.067925048	8.222282469	725.1367967	58.67832452	63.25572395	119.8417417	92.93508625	162.2528355
4-Quinolinecarboxylic acid	97.94990611	301.1242846	266.5496669	198.2109733	139.8368351	131.2174573	111.3873012	97.02832305	99.83357978
5-Hydroxytryptophan	1381.184498	1596.286275	1515.217932	2002.169099	1523.279537	1507.619008	1536.633195	1558.012609	1441.548933
Arginine	14309.17099	6880.671027	6955.385402	8062.736032	2451.841219	3125.606593	3012.86588	3108.346499	3200.655065
Aspartic acid	25.52147634	45.03737926	57.63493323	72.0914627	111.9377272	81.67039073	48.087973	58.73429068	105.946046
Carnosine	339.2096181	296.4822736	286.0275183	347.1374673	197.5199601	122.6914324	876.2717403	9.138337374	1200.679156
Galactosamine	877.0294536	661.7342285	671.0530529	359.2936857	296.1465477	265.2029964	261.685821	236.8864169	222.7820846
Glutamic acid	4391.222289	3237.108515	4450.457521	4525.542721	3834.839649	3181.53492	4352.399165	4059.234834	6179.589747
Glutathione (oxidized)	2984.599905	4877.500262	2891.685992	22718.74607	20972.89806	25269.42093	26715.21599	18227.11623	31599.26951
Glutathione (reduced)	509.6171101	187.6433201	1201.774957	7937.759676	117.3454442	581.3013823	76.21778158	1122.16065	2538.144166
Gly-Val	285.0077046	108.5788705	85.37206065	57.29950715	72.32324076	53.29833213	67.57945186	74.72045275	41.16883161
Histidine	623.8660532	488.5532894	960.5361276	1305.23985	412.1682857	592.3919398	850.1420112	761.7273377	1403.211252
Kynurenine	334.1241684	234.0769091	244.9152237	175.1614282	176.243558	227.9757415	155.3198195	151.3528932	126.3908781
Lysine	1084.937758	883.2106896	361.881903	932.1537396	690.317307	546.9302789	639.4178155	706.1725463	499.8292456
N-Acetylhistidine	200.3924968	277.7039715	190.4867117	21.1115405	39.67537475	16.38198471	20.54403823	28.51171541	3.520582437
1-Methylhistidine	445.3182149	622.8453307	550.1851988	369.7354646	549.0585757	492.5396528	514.2815719	642.4721125	623.2196314
N-Methylisoleucine	16.20035619	492.6020129	1497.270754	122.9556465	277.6058559	1869.1839	987.4903222	155.4295391	41.66494703
N-Methylproline	938.7428732	499.1313592	216.1923532	507.5697667	381.1589578	264.3783459	255.6576448	340.779643	193.5575885
N-Methylserine	152.0224457	160.1800631	159.7586874	286.6155021	226.4782784	234.9162951	278.8110083	291.2046936	352.4399657
Octopine	281.7202152	64.73752135	101.2317286	34.70470102	22.71486929	27.25435883	13.41975415	8.786487509	12.81860358
Ornithine	384.4285182	197.0524491	78.37407494	163.6849489	32.31937027	160.6378494	62.66794813	24.72981174	27.25183324

Proline-hydroxyproline	3556.027665	2771.631333	2832.319433	1986.110328	1673.941468	1730.133215	1442.682233	1548.347592	1568.56843
Propionylcarnitine	1661.842878	538.8093598	662.8899565	1496.382293	305.2174858	628.0526665	1102.642845	512.679592	1942.41749
Pyroglutamic acid	100.1006258	163.21636	132.287034	57.16152577	112.4044713	119.7749039	62.08688045	98.96100444	48.08432192
SDMA	318.8783701	94.22605424	34.12836909	79.3966588	21.17396883	92.43676043	57.72635841	26.10349274	38.2656285
Taurine	11214.04623	14908.26949	11828.0303	12446.82182	11185.03554	9288.065729	10345.40219	9646.755717	13441.90182
Uridine-5-diphosphoacetylglucosamine	1901.679226	7991.789663	4650.909549	39133.04494	11492.20725	10795.85235	11712.83869	8954.683363	24750.45328
Beta-alanine	207.0133653	114.5117631	139.445689	102.7744279	40.79261334	53.13403094	69.10488174	43.30111128	72.23482859
Glycine	209.3990189	329.7188712	412.5213633	849.8603113	383.8905964	825.6283408	614.6295997	498.1858379	840.7471111
Palmitate	423.7109348	206.1394417	440.1949971	1143.412888	222.5175506	374.0186663	104.6679628	157.5507551	719.2376609
Cholate	213515.1134	313897.4281	254887.7721	186200.1355	274746.0158	251519.3518	229606.3552	270284.6536	214835.5433
Glycerophosphocholine	2711.780356	8300.177026	8569.901684	15261.19272	15596.07098	16791.00882	14882.39006	9914.214124	29420.47145
Glutamate	1973.434124	1590.092491	1807.084554	1417.350011	1612.519325	1366.731818	1736.283486	1639.063406	1862.176627
2-Quinolinecarboxylic acid	827.3022575	774.9023857	719.6258357	499.8994055	627.8190298	680.2165966	673.2279005	645.7330828	471.6221786
4-Guanidinobutanoate	244.4659996	398.4163628	600.4269571	1191.126683	240.9732418	858.3407054	383.0137386	236.2894545	385.105423
4-Pyridoxate	378.5446129	494.5432158	492.8691769	392.5494366	478.0967041	635.4637833	521.61866	563.863137	475.4406853
Creatinine	11140.23434	8528.69356	8440.378226	7520.211031	7157.842482	7748.383166	8024.367067	7272.196593	6973.2367
Deoxycarnitine	2303.285317	6075.925129	15690.71506	1510.307167	4228.48748	13297.47769	4851.368978	2698.33444	1324.894146
D-pantothenic acid	57695.69104	59799.66013	70215.83149	23849.24516	25496.42698	24424.55802	35403.45842	32690.87129	47020.59325
Guanosine	26861.23505	19482.30612	25299.73176	20158.0604	21350.14754	21015.9218	17328.91035	20782.05451	25325.90428
Hippurate	5586.641067	4718.074635	4937.740524	4550.143243	4461.488245	4510.065697	4348.480697	3399.827167	3090.357337
Hypoxanthine	35162.60245	24105.55578	39754.56486	42889.6319	15540.83446	14398.17219	14536.6582	13518.9498	33497.11535
Carnitine	4658.327351	4016.717366	7471.237014	942.1477829	3636.460916	3886.953558	3498.914377	3145.93789	3678.662404
Leucine	425495.483	202654.0376	184423.3645	261823.7472	147778.5392	152767.6278	160649.235	138285.8771	171097.8359
Proline	27097.89212	24653.66985	22737.77713	24438.11218	18195.84762	20356.60321	19954.6199	16718.51514	17928.94261
Valine	65738.56839	33191.0299	38358.56552	27196.4081	20825.07925	30270.042	19571.80863	16685.02545	21309.57912
N-Acetyl-D-Tryptophan	123.0062656	265.1156595	242.7324893	481.7485977	380.8519727	366.7517982	401.1034469	447.0977506	422.2694551

Nicotinamide	110093.3282	43588.26787	38018.66993	47953.22432	29676.56595	25730.11523	22285.07265	20547.2098	20766.64776
Phenethylamine	185.2101207	202.0758495	152.6135836	260.9260707	136.4066388	172.6154868	143.702448	82.27718204	124.7341176
Pipecolate	2014.063768	1431.657942	1694.642196	1187.015347	1359.835036	1564.901904	958.5672889	1305.281163	1335.918327
Pyridoxal	1805.25709	1549.357752	1549.553668	1428.382898	1244.529697	1354.262374	1293.226885	1091.661693	1452.24031
Pyridoxine	16755.95656	11885.07693	10657.43665	14901.63224	10043.67721	11199.58201	10918.64877	11182.34696	13749.59246
Cytidine	10129.6891	2771.169535	5899.642119	3785.914149	2486.505553	1822.243435	1689.476874	2392.545003	3415.471162
Citrulline	52.78796067	65.95596929	46.70648373	17.18155493	34.53576592	81.40937148	185.1093801	48.97918437	39.86711651
Inosine	148220.9725	92147.55508	112574.1272	39788.44342	77679.16776	68351.69365	66580.54244	77852.41659	109396.7806
Cytosine	4050.78682	1638.581183	2413.793984	1532.619182	1241.68508	911.7474035	931.6269739	1025.609275	1729.813555
Uridine	19999.64906	9234.42479	14222.87568	7971.33944	11855.84001	12669.41583	9697.803778	10881.84571	17638.01149
Phenylalanine	838.9829588	719.5622855	805.5441701	325.3390943	925.0854173	1277.488777	2030.362467	1414.516436	2335.085091
Uracil	1209.578105	1052.213736	1014.91639	1323.247797	1140.740645	1005.966311	1161.419892	1057.861493	972.5819346
Guanine	9308.009295	5181.637326	9665.842743	11101.98264	4324.62386	3703.735383	4224.304311	4008.014794	10610.92346
Folate	10.89202602	34.96001331	42.0112132	29.24775651	15.68621941	21.10946125	43.28869074	39.56701767	113.4449005
Betaine	6390.901521	3076.404737	3135.578896	2113.258913	2025.620375	2022.835535	1491.568832	1263.746852	1451.849545
Tryptophan	206.0804716	179.3156128	204.8582532	454.5633912	395.2196114	739.7845624	1014.563422	707.2636299	1340.404042
Pyroglutamate	45233.22281	29131.15599	32144.24454	61143.09082	31712.4687	48090.69179	44850.55616	38379.38521	48686.45472
4-Acetamidobutanoate	223.0084377	76.23531058	165.5569575	30.97524963	38.48243653	45.05211898	30.10827248	27.80312739	32.04345996
Adenosine	42387.09258	36751.10138	45328.61208	12699.66001	27948.87566	50093.74865	35648.16845	42380.52378	61129.56515
2-Hydroxyphenylacetate	807.274657	1001.634187	755.8274644	770.0913978	789.6459269	771.325078	830.1019759	719.0268183	643.0141692
Lipoamide	1002.500927	1124.570787	1065.237377	818.4216904	797.0605886	978.7191671	919.5349414	871.1759716	827.5399257
Suberate	1658.342584	1942.533706	2051.218112	1684.570705	1745.202409	2462.05239	2384.926109	1937.226924	1540.269107
4-Methylcatechol	203.8197906	187.7604063	246.7151482	293.7176981	211.3613777	276.6223376	365.7266195	266.6290479	394.8797507
